# Antiplatelet Therapy Continuation Versus Discontinuation in Patients Undergoing Dental Extraction: A Systematic Review

**DOI:** 10.7759/cureus.117834

**Published:** 2026-10-13

**Authors:** Piratiba A, Karthikeyan G R, Balaguhan Balasubramanian, Indrapriyadharshini Karthikeyan, Revanth MP

**Affiliations:** 1 Department of Oral and Maxillofacial Surgery, Karpaga Vinayaga Institute of Dental Sciences, Madhuranthagam, IND; 2 Department of Public Health Dentistry, Karpaga Vinayaga Institute of Dental Sciences, Madhuranthagam, IND

**Keywords:** antiplatelet agents, dental care, hemorrhage, platelet aggregation inhibitors, tooth extraction

## Abstract

Antiplatelet medications are widely prescribed for the prevention of cardiovascular and cerebrovascular thrombotic events; however, their continuation during dental extraction remains a clinical concern because of the potential for postoperative bleeding. This systematic review evaluated the available randomized controlled evidence on whether antiplatelet therapy should be discontinued before dental extraction. A systematic literature search was conducted in PubMed/MEDLINE, the Cochrane Library, Scopus, Web of Science, and Google Scholar for studies published between January 2000 and July 2026. Randomized controlled trials (RCTs) involving patients receiving antiplatelet therapy who underwent dental extraction and comparing continuation with discontinuation of treatment were eligible. Two reviewers independently screened the studies, extracted the data, and assessed the risk of bias using the Cochrane Risk of Bias 2 tool. Seven RCTs involving 646 participants were included in the qualitative synthesis. The included studies evaluated aspirin and/or clopidogrel therapy and reported interruption periods ranging from 72 hours to seven days. Four studies reported no postoperative bleeding in either treatment group, whereas the remaining studies reported only mild or limited postoperative oozing. Bleeding events were generally managed successfully with local hemostatic measures, including pressure application, suturing, and local hemostatic materials. Six studies had some concerns regarding the risk of bias, and one was judged to have a high risk of bias. Owing to heterogeneity in antiplatelet regimens, extraction procedures, bleeding definitions, and assessment time points, a quantitative meta-analysis was not performed. Available evidence suggests that continuation of antiplatelet therapy during dental extraction is generally associated with manageable postoperative bleeding, although the certainty of evidence remains limited.

## Introduction and background

Cardiovascular disease (CVD) is a major cause of morbidity and mortality worldwide. Antiplatelet therapy reduces thrombotic events in patients with cardiovascular and cerebrovascular disorders [1]. These agents are commonly prescribed for myocardial infarction, angina, ischemic heart disease, and cerebrovascular diseases. Aspirin, clopidogrel, ticlopidine, and prasugrel are used as single-agent or combination therapy [2]. Aspirin irreversibly inhibits cyclooxygenase-1 and thromboxane A2 synthesis, whereas clopidogrel, ticlopidine, and prasugrel inhibit platelet aggregation by blocking platelet adenosine diphosphate receptors [3]. Because these effects persist for the lifespan of circulating platelets, antiplatelet therapy can prolong bleeding time and influence perioperative hemostasis.

Dental extraction is a common minor surgical procedure in patients receiving antiplatelet medication. The possibility of prolonged postoperative bleeding has traditionally led clinicians to consider temporary interruption of therapy before extraction [4]. However, discontinuation may expose patients to an increased risk of thromboembolic complications, particularly those with CVD [5]. Perioperative management, therefore, requires balancing the risk of bleeding against the consequences of interrupting medication prescribed to prevent vascular events [6].

Earlier recommendations have suggested stopping aspirin before invasive procedures based on platelet lifespan, while other evidence supports the continuation of antiplatelet therapy for minor oral procedures [7,8]. Studies evaluating single- and dual-antiplatelet regimens have reported that postoperative bleeding, when present, is generally limited and can often be controlled using local hemostatic measures such as pressure application, suturing, and hemostatic materials [9,10]. Nevertheless, variations in clinical recommendations and differences in study designs, antiplatelet regimens, extraction procedures, and bleeding assessment methods have contributed to uncertainty regarding treatment interruption. Therefore, this systematic review aimed to evaluate whether cessation of antiplatelet therapy before dental extraction is necessary by comparing postoperative bleeding outcomes between patients who continued and those who discontinued antiplatelet therapy.

## Review

This systematic review was conducted and reported in accordance with the Preferred Reporting Items for Systematic Reviews and Meta-Analyses (PRISMA) 2020 guidelines [11]. The review question was structured using the Population, Intervention, Comparison, Outcome, and Studies (PICOS) framework. The population comprised patients receiving antiplatelet therapy and undergoing dental extraction; the intervention was continuation of antiplatelet therapy, the comparison was discontinuation before extraction, the primary outcome was postoperative bleeding, and the studies included randomized controlled trials (RCTs) [12].

A systematic literature search was conducted in PubMed/MEDLINE, the Cochrane Library, Scopus, Web of Science, and Google Scholar for studies published between January 2000 and July 2026. The search strategy combined medical subject headings, terms, and free-text keywords related to antiplatelet therapy, dental extraction, and bleeding. PubMed/MEDLINE included terms such as “Platelet Aggregation Inhibitors,” aspirin, “acetylsalicylic acid,” antiplatelet, and clopidogrel; “Tooth Extraction,” “Oral Surgical Procedures,” “dental extraction,” “tooth extraction,” and “tooth removal”; and “Hemorrhage,” bleeding, “postoperative bleeding,” and “bleeding complications.” Terms describing continuation or interruption of therapy, including continuation, discontinue, stop, suspend, maintain, therapy, and management, were also incorporated using the Boolean operators AND and OR. The searches were restricted to English-language human studies published during a predefined period.

RCTs involving patients receiving antiplatelet therapy and undergoing dental extractions were eligible. Studies were required to compare continuation with discontinuation of antiplatelet therapy and to report postoperative bleeding or related hemostatic outcomes. Non-randomized studies, case reports, case series, reviews, studies involving healthy individuals or other anticoagulant therapies, non-human studies, and articles published in languages other than English were also excluded.

Two reviewers (PA and BB) independently screened titles and abstracts, followed by a full-text assessment of potentially eligible studies. Duplicates were removed, and disagreements were resolved through discussion and consensus. The study selection process was documented using the PRISMA flow diagram. Seven RCTs fulfilled the eligibility criteria and were included in the final qualitative synthesis. Data were extracted, including author and publication year, sample size, participant characteristics, antiplatelet regimen, intervention and comparison groups, type of dental procedure, duration of treatment interruption, postoperative bleeding outcomes, bleeding assessment methods, and local hemostatic measures.

The risk of bias was assessed independently using the Cochrane Risk of Bias 2 (RoB 2) tool for randomized trials [13]. Two reviewers independently assessed the risk of bias of all included RCTs. Any disagreements between the reviewers were resolved through discussion and consensus; when consensus could not be reached, a third reviewer was consulted. The assessment covered bias arising from the randomization process, deviations from intended interventions, missing outcome data, outcome measurement, and selection of reported results. Each domain was classified as low risk, some concerns, or high risk of bias. The findings were synthesized narratively because the included studies differed in antiplatelet regimens, treatment-interruption intervals, extraction procedures, bleeding definitions, and outcome-assessment time points. Although three studies reported sufficiently comparable numerical data, quantitative synthesis was not performed because the limited number of directly comparable studies would provide a relatively unstable pooled estimate. Therefore, this review focused on a qualitative comparison of postoperative bleeding and its management following continuation or discontinuation of antiplatelet therapy.

Results

The systematic search identified 335 records across PubMed/MEDLINE, the Cochrane Library, Scopus, Web of Science, and Google Scholar. After the removal of 90 duplicate records, 245 records remained for title and abstract screening. Of these, 216 were excluded because they did not meet the predefined eligibility criteria. The remaining 29 full-text reports were assessed for eligibility, of which 22 were excluded for reasons including nonrandomized study design, missing data, and improper grouping. Consequently, seven RCTs were included in the qualitative synthesis (Figure 1).

**Figure 1:**
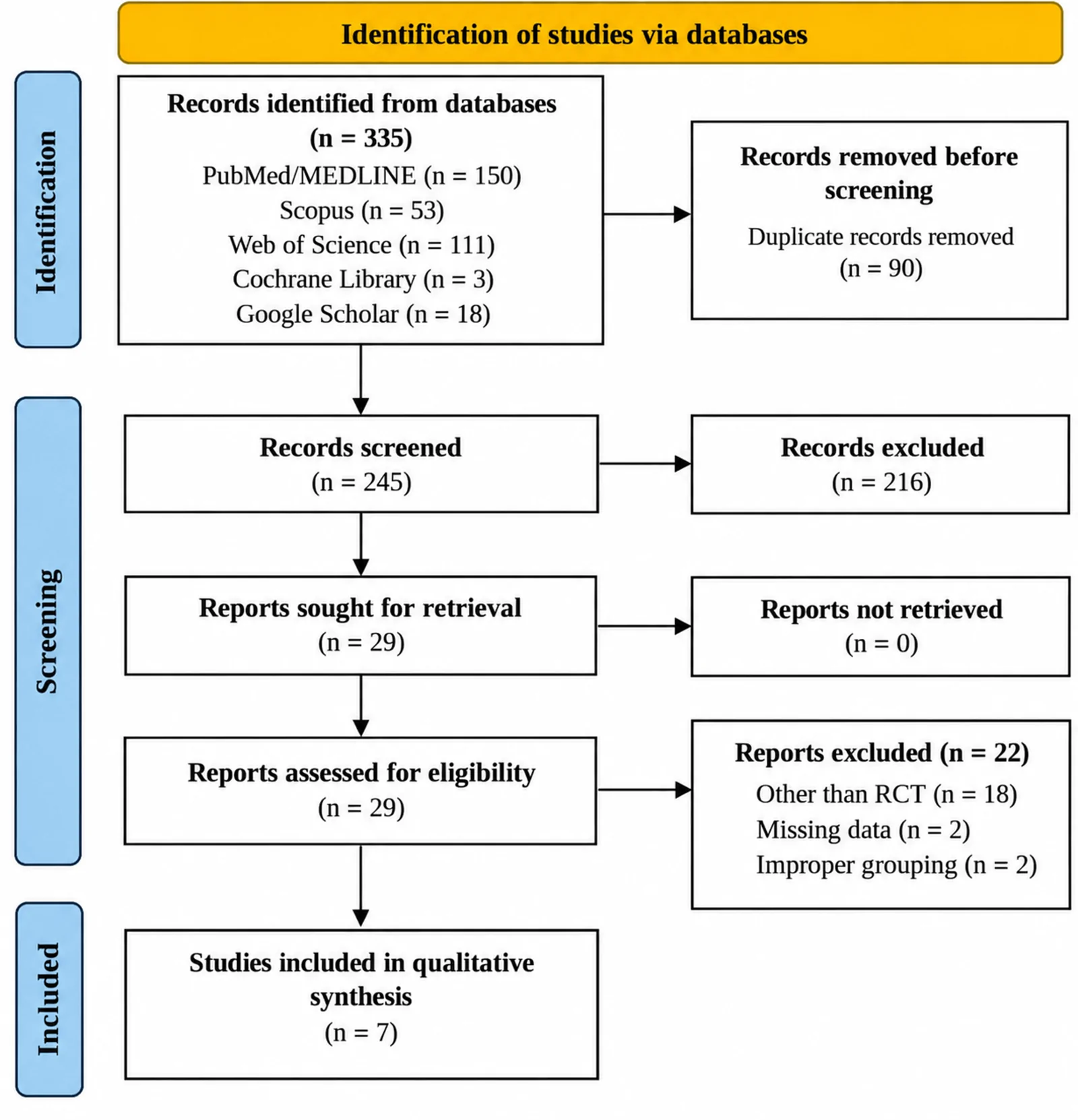
PRISMA flow diagram Image created with Canva software (Canva, Pvt. Ltd., Australia). PRISMA, Preferred Reporting Items for Systematic Reviews and Meta-Analyses; RCT, randomized controlled trial

The included studies, published between 2000 and 2020, comprised 646 participants according to the study data [9,10,14-18]. The studies evaluated patients receiving aspirin or clopidogrel who had undergone dental extraction or minor oral surgical procedures. The continuation group generally maintained antiplatelet therapy until the time of extraction, whereas the discontinuation group interrupted therapy for 72 h to seven days before the procedure. The sample sizes ranged from 39 to 190 participants. The characteristics of the included studies are shown in Table 1.

**Table 1:** Characteristics of the included studies

Author, year	Sample size, N	Antiplatelet regimen	Dental procedure	Continuation, n (%)	Discontinuation, n (%)	Interruption
Ardekian et al. [10], 2000	39	Aspirin	Dental extraction	19 (48.7)	20 (51.7)	7 days
Medeiros et al. [9], 2011	63	Aspirin	At least one molar extraction	31 (49.2)	32 (50.8)	7 days
Gupta et al. [14], 2017	44	Aspirin	Dental extraction	25 (56.8)	19 (43.2)	7 days
Varghese et al. [15], 2015	190	Antiplatelet therapy	Single molar extraction	95 (50.0)	95 (50.0)	5 days
Dinkova et al. [16], 2017	130	Aspirin/clopidogrel	Single or multiple extraction	65 (50.0)	65 (50.0)	72 hours
Malik et al. [17], 2020	100	Aspirin/clopidogrel	Third molar extraction	50 (50.0)	50 (50.0)	5 days
Eapen et al. [18], 2017	80	Aspirin	Atraumatic extraction	40 (50.0)	40 (50.0)	5 days

Across the included studies, postoperative bleeding was generally absent or limited to mild transient oozing. Ardekian et al. [10], Medeiros et al. [9], Gupta et al. [14], and Eapen et al. [18] reported no postoperative bleeding in either the continuation or discontinuation groups. Varghese et al. [15] observed slightly more early postoperative oozing among patients who continued antiplatelet therapy; however, bleeding was limited and resolved during follow-up. Similarly, Dinkova et al. [16] reported comparable bleeding frequencies between the continuation and discontinuation groups at the assessed postoperative time points. Malik et al. [17] also reported only mild postoperative oozing, with no clinically significant or uncontrolled hemorrhage. The detailed bleeding outcomes are summarized in Table 2.

**Table 2:** Postoperative bleeding outcomes reported in the included studies

Author, year	Continuation group	Discontinuation group	Main observation
Ardekian et al. [10], 2000	No bleeding	No bleeding	No postoperative bleeding reported.
Medeiros et al. [9], 2011	No bleeding	No bleeding	No postoperative bleeding reported.
Gupta et al. [14], 2017	No bleeding	No bleeding	No postoperative bleeding reported.
Varghese et al. [15], 2015	11.5% at 1 h; 6.3% at 24 h	8.4% at 1 h; 2.1% at 24 h	No active bleeding at 48 h or 5 days; local measures used.
Dinkova et al. [16], 2017	23.5% at 10 min; 5.9% at 10-30 min; 1.5% at 12-24 h	26.5% at 10 min; 5.9% at 10-30 min; 1.5% at 12-24 h	Bleeding/oozing decreased over time.
Malik et al. [17], 2020	4% at 1 h; 6% at 24 h	2% at 1 h; 4% at 24 h	Mild ooze reported; no active bleeding described.
Eapen et al. [18], 2017	No postoperative bleeding	No postoperative bleeding	No postoperative bleeding reported.

When bleeding occurred, it was generally managed using conventional local hemostatic measures, including pressure application, suturing, and placement of local hemostatic materials. None of the included studies reported clinically significant uncontrolled hemorrhage requiring major intervention. The methods used for bleeding assessment and hemostatic management are shown in Table 3.

**Table 3:** Bleeding assessment and local hemostatic management

Study	Assessment time points	Reported bleeding pattern	Hemostatic management
Ardekian et al. [10], 2000	Intraoperative and 1 week postoperative	No postoperative bleeding	Not required.
Medeiros et al. [9], 2011	Post-extraction follow-up; exact time point not reported	No bleeding	Not required.
Gupta et al. [14], 2017	30 min, 24 h, 48 h, 72 h, and 1 week	No excessive postoperative bleeding	Not required.
Varghese et al. [15], 2015	1, 24, 48 h; 5 days	Mild early oozing	Local hemostatic measures.
Dinkova et al. [16], 2017	10 min; 10-30 min; 12-24 h	Early bleeding/oozing declining over time	Local measures as required.
Malik et al. [17], 2020	1 and 24 h	Mild ooze; no active bleeding	Local measures as required.
Eapen et al. [18], 2017	12 h and 24 h	No postoperative bleeding	Not required.

Risk of bias assessment using the Cochrane Risk of Bias 2 tool showed that six studies were judged to have some concerns, and one study was judged to have a high risk of bias. Concerns were primarily related to aspects of the randomization process, allocation concealment, and selection of the reported results. The short follow-up periods and low frequency of clinically important bleeding events limited the assessment of some potential sources of bias. The overall risk of bias assessment is presented in Figure 2.

**Figure 2:**
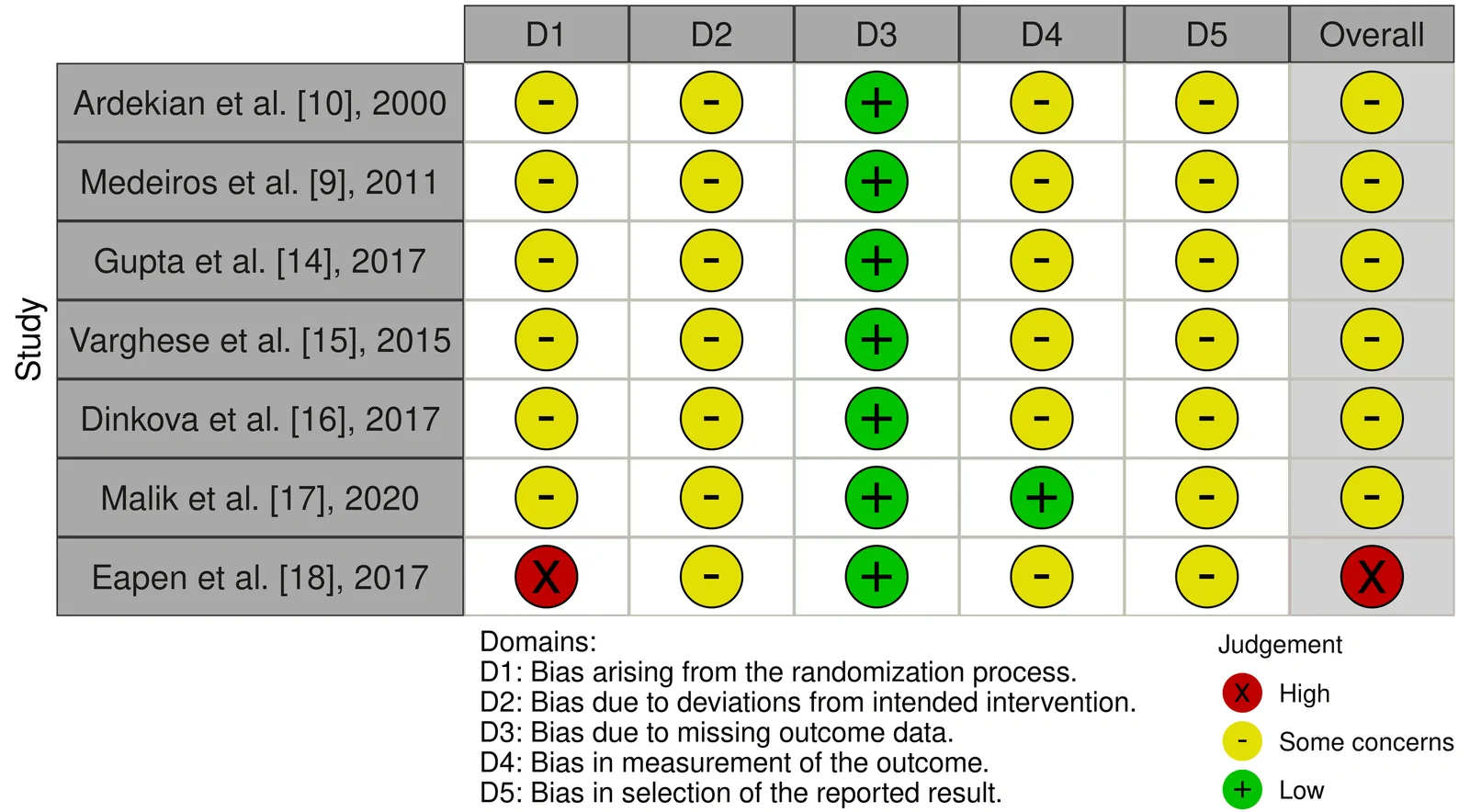
Overall risk-of-bias assessment of the included randomized controlled trials using the Cochrane RoB 2 tool RoB 2, Risk of Bias 2

As the included studies differed in antiplatelet regimens, duration of treatment interruption, extraction procedures, definitions and timing of bleeding assessment, and reporting methods, the findings were synthesized narratively rather than quantitatively. Although three trials (Varghese, Dinkova, and Malik) reported extractable numerical bleeding-event data, their assessment intervals were not sufficiently harmonized for direct quantitative pooling: Varghese et al. [15] and Malik et al. [16] reported outcomes at one and 24 hours, whereas Dinkova et al. [17] assessed bleeding at earlier intervals and over a 12-24-hour period. A summary of the principal findings of the included studies is presented in Table 4.

**Table 4:** Summary of findings from the systematic review

Domain	Summary
Included evidence	Seven randomized controlled trials were included in the qualitative synthesis.
Total sample	646 participants were reported in the manuscript.
Intervention	Continuation of antiplatelet therapy during dental extraction or minor oral surgical procedures.
Comparison	Temporary discontinuation of antiplatelet therapy before the procedure.
Bleeding outcome	Most studies reported no bleeding or only mild postoperative oozing.
Hemostatic management	Reported bleeding was generally managed with local hemostatic measures.
Synthesis	Narrative synthesis was used because of clinical and methodological heterogeneity and limited directly comparable numerical data.

Overall, the included RCTs consistently reported that postoperative bleeding following dental extraction was absent or mild and could be controlled with local hemostatic measures, including among patients who continued antiplatelet therapy. However, the small number of eligible trials and methodological concerns identified in several studies should be considered when interpreting the evidence.

Using the GRADE (Grading of Recommendations Assessment, Development and Evaluation) framework, the certainty of evidence for postoperative bleeding was rated as low. The certainty was downgraded because most trials had methodological concerns, the studies varied in antiplatelet regimens, extraction procedures, bleeding definitions, and assessment time points, and the limited number of clinically important bleeding events precluded precise estimation of the comparative effect (Table 5).

**Table 5:** GRADE assessment of the certainty of evidence for postoperative bleeding GRADE certainty: ⊕⊕⊕⊕ high, ⊕⊕⊕◯ moderate, ⊕⊕◯◯ low, ⊕◯◯◯ very low. Certainty was downgraded for serious risk of bias, inconsistency in bleeding definitions and assessment procedures, and imprecision resulting from the small evidence base, infrequent clinically important bleeding events, and absence of a pooled effect estimate. RCT, randomized controlled trial; GRADE, Grading of Recommendations Assessment, Development and Evaluation

Outcome	Studies (participants)	Risk of bias	Inconsistency	Indirectness	Imprecision	Publication bias	Overall certainty	Summary of findings
Postoperative bleeding	7 RCTs (646)	Serious ↓1	Serious ↓1	Not serious	Serious ↓1	Undetected	Low ⊕⊕◯◯	Continuation of antiplatelet therapy was generally associated with no bleeding or mild, controllable postoperative bleeding. No study reported clinically significant uncontrolled hemorrhage requiring major intervention.

Discussion

The present systematic review evaluated whether discontinuation of antiplatelet therapy before dental extraction is necessary by synthesizing evidence from seven RCTs involving 646 participants. Overall, the included studies demonstrated that continuation of antiplatelet therapy was generally associated with either no postoperative bleeding or only mild, self-limiting bleeding that could be controlled using conventional local hemostatic measures. These findings indicate that clinically significant uncontrolled hemorrhage is uncommon among the patients evaluated in the included trials.

The absence of substantial differences in clinically important bleeding may be explained by the nature of dental extraction itself and the effectiveness of local hemostatic measures. Dental extraction produces a localized wound in a highly vascular region; however, the formation of a stable clot can be supported by mechanical compression, suturing, and local hemostatic agents [19]. Consequently, pharmacological inhibition of platelet function may increase the tendency for minor oozing without necessarily resulting in prolonged or clinically significant hemorrhage. This pattern was particularly evident in the studies by Varghese et al. [15] and Malik et al. [17], in which small differences in early bleeding were reported between the treatment groups, but the bleeding remained mild and did not progress to uncontrolled hemorrhage. Similar findings were reported by Dinkova et al. [16], who observed comparable bleeding frequencies between the continuation and discontinuation groups at the assessed postoperative intervals.

These findings are also consistent with the broader clinical literature suggesting that antiplatelet-associated bleeding during dental procedures is usually manageable using local measures. Previous systematic reviews have reported that continuation of single or dual antiplatelet therapy generally does not result in clinically significant bleeding following dental procedures, whereas interruption of therapy may expose patients to thrombotic complications [4,20]. Maintaining antiplatelet therapy is particularly relevant because these drugs are prescribed to reduce the risk of cardiovascular and cerebrovascular events [1]. Therefore, the decision to interrupt therapy cannot be based solely on the possibility of minor postoperative bleeding and should consider the indication for antiplatelet treatment and the patient's underlying thrombotic risk [9].

From an evidence perspective, this review has the advantage of including only RCTs, thereby focusing on comparative evidence rather than relying predominantly on retrospective or observational studies. Nevertheless, the level of evidence should be interpreted cautiously. Although RCTs generally provide stronger evidence for comparative treatment effects, six of the seven included studies were judged to have some concerns regarding the risk of bias [9,10,14-17], whereas one study was judged to have a high risk of bias [18]. Concerns involved aspects of randomization, allocation concealment, and selection of reported outcomes. Furthermore, the relatively small number of eligible trials limited the precision and generalizability of the conclusions. Therefore, this review provides suggestive randomized evidence rather than definitive, high-certainty evidence.

Several deficiencies in the available evidence should be recognized. The included studies differed substantially in the antiplatelet regimens evaluated, including aspirin and clopidogrel, the duration of treatment interruption, and the types and complexity of dental extractions. The definitions of postoperative bleeding and the assessment time points were inconsistent. Previous systematic reviews have similarly highlighted the heterogeneity in antiplatelet regimens, dental procedures, and bleeding outcomes across the available literature [4,20]. These differences prevented reliable quantitative comparisons across all studies and were the principal reason for adopting a narrative synthesis rather than a meta-analysis. In addition, the available trials generally involved relatively short follow-up periods and were primarily designed to evaluate bleeding rather than thrombotic outcomes after treatment interruption. Thus, the present review cannot adequately quantify the potential cardiovascular or cerebrovascular consequences of discontinuing antiplatelet therapy.

Clinical considerations for continuation or interruption of antiplatelet therapy

From a clinical perspective, the decision to continue or interrupt antiplatelet therapy should be individualized according to the indication for antiplatelet treatment, thrombotic risk, type and extent of the dental procedure, and anticipated bleeding risk. For routine or limited dental extractions, particularly when effective local hemostatic measures can be applied, continuation of antiplatelet therapy is generally supported by the findings of the present review because the reported bleeding was predominantly mild and controllable. Patients receiving antiplatelet therapy for established cardiovascular or cerebrovascular disease may have a substantial thrombotic risk if treatment is interrupted; therefore, discontinuation should not be undertaken routinely or solely because of the anticipated possibility of postoperative bleeding [1]. For procedures associated with greater tissue trauma or multiple and complex extractions, bleeding risk should be assessed individually, with consideration of staged treatment and appropriate local hemostatic measures; any proposed modification of antiplatelet therapy should be undertaken only after consultation with the patient's treating physician or cardiologist [21]. Interruption of antiplatelet therapy, when considered necessary because of an exceptionally high procedural bleeding risk, should occur only after assessment of the patient's underlying thrombotic risk and in consultation with the prescribing physician. The evidence synthesized in this review does not establish a universal threshold at which antiplatelet therapy should be discontinued, particularly because the included trials varied in drug regimens, interruption durations, and procedural complexity.

Clinical implications, limitations, and future recommendations

Clinically, the findings indicate that routine discontinuation of antiplatelet therapy before uncomplicated dental extraction should not be considered solely on the basis of anticipated bleeding, as most bleeding events reported in the included trials were mild and manageable with local hemostatic measures [22]. However, treatment decisions should remain individualized, particularly in patients receiving antiplatelet therapy for important cardiovascular indications or those undergoing more extensive oral surgery. Any alteration in prescribed antiplatelet therapy should involve appropriate medical consultation rather than unilateral discontinuation.

The principal limitations of this review were the small number of eligible RCTs, methodological concerns within most included studies, heterogeneity in antiplatelet regimens and surgical procedures, inconsistent definitions of bleeding, and insufficient assessment of thrombotic outcomes. Future research should include larger, well-designed multicenter RCTs using standardized definitions and objective bleeding scales, clearly defined extraction protocols, longer follow-up periods, and systematic reporting of both bleeding and thrombotic outcomes. Future studies should also distinguish between single and dual antiplatelet therapy and evaluate patients with different levels of surgical complexity. Such evidence would allow a more precise estimation of the balance between bleeding risk and the potential consequences of antiplatelet interruption.

## Conclusions

This systematic review found that continuation of antiplatelet therapy during dental extraction was generally associated with no bleeding or mild, controllable postoperative bleeding. However, the limited number of RCTs, methodological concerns, and clinical heterogeneity reduce the certainty of the overall evidence. Routine interruption should therefore not be based solely on concerns about minor bleeding, and treatment decisions should consider individual thrombotic risk and procedural complexity. Larger, standardized prospective trials are required to strengthen the evidence base.
